# Effects of Different Slope Aspects on Leaf Non-Structural Carbohydrate Characteristics and Leaf–Soil Stoichiometry of *Sapindus mukorossi*

**DOI:** 10.3390/plants14203131

**Published:** 2025-10-11

**Authors:** Heng Wang, Chengyao Liu, Dingming Wei, Yunbin Zhou, Tingwen He, Tangjie Zhao, Chengbo Peng, Lianchun Wang, Yuan Zheng

**Affiliations:** 1 College of Southwest Forestry University Forestry Southwest Key Laboratory of Conservation and Utilization of Mountain Forest Resources, Ministry of Education, Kunming 650224, China; 2 Urban Design Institute, Southwest Forestry University, Kunming 650224, China; 3 College of Biology and Food Engineering, Southwest Forestry University, Kunming 650224, China

**Keywords:** *Sapindus mukorossi*, slope aspect, slope position, non-structural carbohydrate, leaf–soil stoichiometry

## Abstract

Slope aspect and slope position have an important influence on plant growth by changing the microclimate and soil conditions such as light, temperature, moisture, and nutrients. In this study, 15-year-old *Sapindus mukorossi* forests with different slope aspects and positions were selected and the differences in tree height and diameter at breast height (DBH), leaf non-structural carbohydrate (NSC) characteristics, and leaf–soil nitrogen (N), phosphorus (P), and potassium (K) stoichiometric characteristics between sunny and shady slopes, and upper, middle, and down slope positions were compared and analyzed. The results show that the tree height and DBH of *S. mukorossi* were better in the same slope aspect and lower slope position, while in the same slope position, the tree height and diameter at DBH were better on the shady slopes. In the upper slope position, the starch content on the shady slope was significantly higher than that on the sunny slope, and the NSC content was significantly higher than that on the sunny slope. On shady and sunny slopes, *S. mukorossi* is mainly limited by N. The leaf and soil P content of *S. mukorossi* on the sunny slope was the highest and significantly higher than that on the upper slope. The coefficient of variation of each index of *S. mukorossi* on the shady slope and the sunny slope was medium and below. Soil N/P, soil N, soil N/K, soluble sugar/starch, leaf P, leaf K, leaf N, and soil K had strong plasticity under different slope aspects. Therefore, it indicated that the shady slope and down slope were more suitable for *S. mukorossi*.

## 1. Introduction

Topography is an important factor affecting the distribution of forests. It plays an important role in the habitat diversity of plant communities by affecting soil moisture, nutrients, and microclimate conditions [1,2,3]. Slope aspect is an important topographic factor affecting plant growth, and different slope aspects can be divided into different slope positions. These factors jointly affect the redistribution of light, water, and soil nutrients required for plant growth [4]. In recent years, many scholars have studied the relationship between terrain factors and soil fertility and forest growth. Studies have shown that soil nutrients under *Pinus tabulaeformis* and *Pinus sylvestris* var. *mongolica* forests are concentrated in the relatively low slope position, indicating that down slope positions can accumulate more nutrients needed for plant growth [5]. Other studies have compared and analyzed the tree growth and soil physical and chemical properties of *Armeniaca sibirica* at different slope positions, and it was found that with the decline of slope position, the tree growth status, and soil physical and chemical properties gradually improved [6]. Tree height and DBH are the most basic morphological characteristics of tree growth and development, which can intuitively reflect the adaptability of trees to changes in the external environment [7]. Slope aspect can change soil conditions and hydrothermal conditions, resulting in differences in plant growth [8,9]. The slope position affects the soil water content, and the water content gradually decreases from the down slope position to the upper slope position, while the plant growth is closely related to the soil water content [10].

Non-structural carbohydrates (NSCs) include soluble sugar and starch and their concentration and variation are key indicators that affect plant physiological activities and responses to changes in the external environment, playing an important role in maintaining the normal growth of plants and resisting the stress of external environmental factors [11]. Ecological chemometrics has become an important method to study plant nutrient use efficiency, nutrient limitation, population dynamics, and plant response to global climate change [12]. Nitrogen (N), phosphorus (P), and potassium (K) are important elements in plant growth and development, which are closely related to plant growth, metabolism, and various physiological activities [13]. Leaves are the main photosynthetic organs of plants and source of energy for trees and are very sensitive to environmental changes [14]. The content of nutrient elements in leaves is related to many key functions of plant growth and development and ecosystems and can be used as an indicator to evaluate plant nutrient utilization and response to environmental changes [15]. Therefore, exploring the changing patterns of NSCs and N, P, and K stoichiometry in plant leaves is an ideal entry point for understanding the response of plant growth and ecophysiological processes to multi-factor environmental changes and adaptations. At the same time, N, P, and K are also important indicators of soil fertility and land productivity [16]. N, P, and K in soil, as a large proportion of nutrients required for plant growth, are an important core of nutrient element cycling [17]. They are of great significance for exploring the cycling of nutrient elements in the soil cycle and the change in ecological environments, for rationally utilizing soil resources, for creating the best ecological benefits, for preventing soil fertility decline, and for improving the ecological environment.

*Sapindus mukorossi* is a tall deciduous tree widely distributed in eastern, southern, and southwestern China. Saponin extracted from bark has a variety of functions, including cleaning and disinfection, heavy metal dissociation, and antibacterial and antitumor properties, and has important application value in the fields of biological, daily chemical, biomedical, and ecological restoration [18,19,20,21]. At present, the research on *S. mukorossi* mainly focuses on breeding, diseases and insect pests, environmental stress, and other aspects, while the research on the relationship between topographic factors and *S. mukorossi* is relatively limited [19,22,23]. We hypothesize on the influence of different slope aspects and positions on the presence of *S. mukorossi*. In this study, an *S. mukorossi* plantation was used as the research object to study the growth, leaf NSC, and leaf and soil N, P, and K stoichiometry of *S. mukorossi* under different slope aspects and slope positions, in order to explore the suitable terrain factors for the growth of the *S. mukorossi* plantation, and provide reference for improving the growth and afforestation efficiency of *S. mukorossi*.

## 2. Results

### 2.1. Effects of Slope Aspect and Position on Tree Height and DBH of S. mukorossi

**Figure 1:**
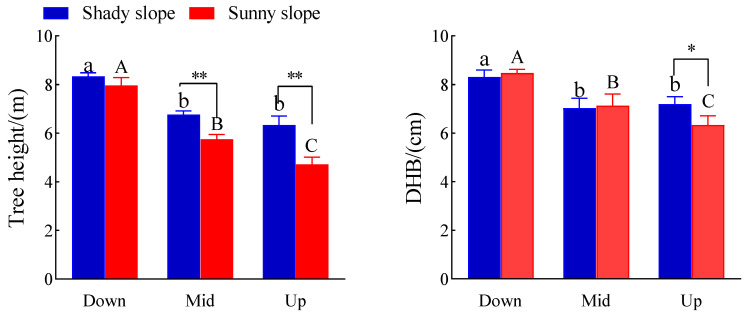
Effects of slope aspect and position on tree height and DBH of *S. mukorossi* plantation. Note: different lower case letters in the figure indicate significant difference between different slope positions on the shady slope (*p* < 0.05), and different majuscule indicate significant difference between different slope positions on the sunny slope (*p* < 0.05); * indicates significant difference between different aspects in the same slope position (*p* < 0.05), ** indicates extremely significant difference between different aspects in the same slope position (*p* < 0.01). The same as below.

Figure 1 shows that different slope aspects and positions have different effects on the height and DBH of *S. mukorossi* (*p* < 0.05). The tree height of *S. mukorossi* on shady and sunny slopes reached a maximum at the down slope position, which was 8.33 and 7.97 m, respectively. On the shady slope, the height of *S. mukorossi* was 18.7% and 24.0% less on the middle and upper slope than that on the down slope, respectively, while on the sunny slope, it was 27.9% and 40.8% less on the middle and upper slope than that on the down slope, respectively. In the middle and upper slope positions, the height of *S. mukorossi* on the shady slope was 17.7% and 34.1% higher than that on the sunny slope (*p* < 0.01). The DBH of *S. mukorossi* on shady and sunny slopes reached a maximum of 8.30 cm and 8.47 cm on the down slope, while the DBH of *S. mukorossi* on the shady slope was 15.3% and 13.3% less on the middle and upper slopes than that on the down slope The DBH of *S. mukorossi* on the sunny slope was 15.8% and 25.3% less than that on the middle slope and upper slope, respectively, while it was 13.7% higher on the shady slope than that on the sunny slope. The results showed that *S. mukorossi* had a better tree height and DBH on the down slope and shady slope.

### 2.2. Effects of Slope Aspect and Position on Leaf NSC Characteristics of S. mukorossi

**Figure 2:**
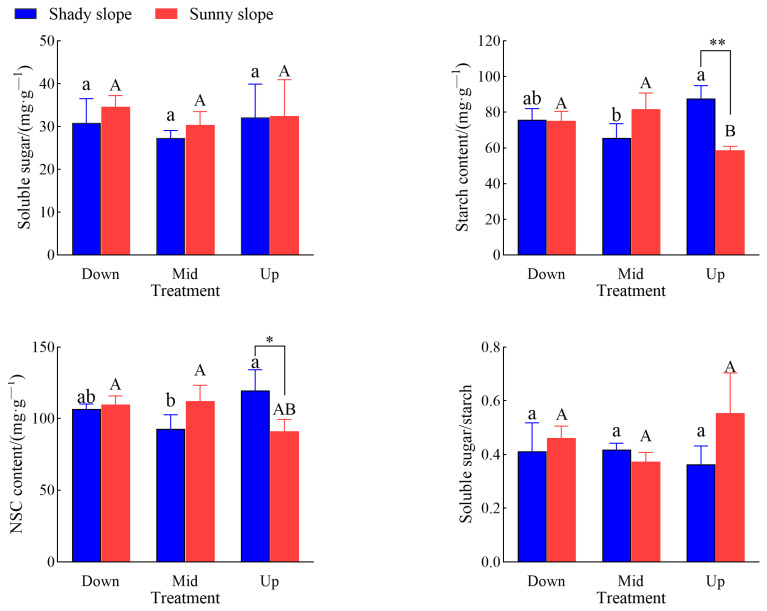
Effects of slope aspect on NSCs and its components in leaves of *S. mukorossi.* Note: different lower case letters in the figure indicate significant difference between different slope positions on the shady slope (*p* < 0.05), and different majuscule indicate significant difference between different slope positions on the sunny slope (*p* < 0.05); * indicates significant difference between different aspects in the same slope position (*p* < 0.05), ** indicates extremely significant difference between different aspects in the same slope position (*p* < 0.01).

Figure 2 shows that there was no significant difference in the soluble sugar content of *S. mukorossi* leaves with slope aspect and slope position. The highest starch content in leaves of *S. mukorossi* was 87.6 mg/g on the upper slope, which was 33.7% higher than that on the middle slope (*p* < 0.05), while the highest starch content was 81.7 mg/g on the middle slope and the sunny slope, in the upper slope position, while the starch content in the leaves of *S. mukorossi* on the shady slope was significantly higher than that on the sunny slope by 49.2% (*p* < 0.01). The highest NSC content in leaves of *S. mukorossi* was 119.7 mg/g on the upper slope, which was 28.8% higher than that on the middle slope. There was no significant difference in leaves of *S. mukorossi* on the sunny slope, while the NSC content in leaves of *S. mukorossi* on the shady slope was 31.2% higher than that on the sunny slope. The ratio of soluble sugar to starch in leaves of *S. mukorossi* had no significant difference with slope aspect and slope position.

### 2.3. Effects of Slope Aspect and Position on Leaf Stoichiometry of S. mukorossi

**Figure 3:**
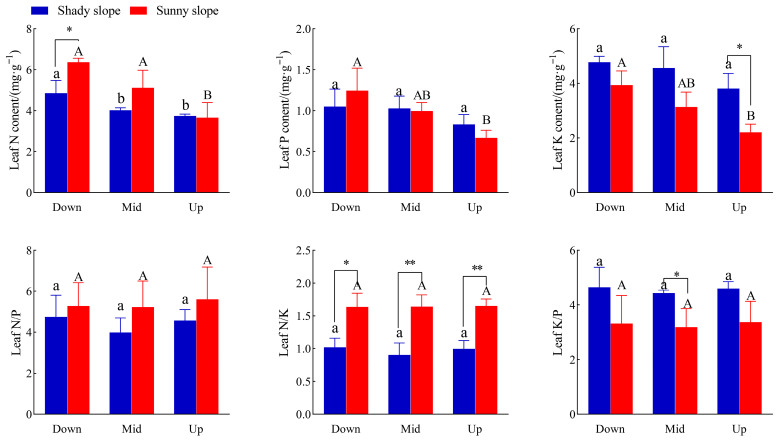
Effects of different slope aspects on leaf stoichiometry of *S. mukorossi.* Note: different lower case letters in the figure indicate significant difference between different slope positions on the shady slope (*p* < 0.05), and different majuscule indicate significant difference between different slope positions on the sunny slope (*p* < 0.05); * indicates significant difference between different aspects in the same slope position (*p* < 0.05), ** indicates extremely significant difference between different aspects in the same slope position (*p* < 0.01).

From Figure 3, the highest N content of *S. mukorossi* was 4.86 mg/g in the down slope position, which was significantly higher than that in the middle and upper slope positions by 20.9 and 29.9% (*p* < 0.05), respectively; however, the contents of P and K in leaves of *S. mukorossi* on the shady slope did not change significantly with slope position. The contents of N, P, and K in leaves of *S. mukorossi* on the sunny slope were 6.37 mg/g, 1.24 mg/g, and 3.94 mg/g on the down slope, which were 74.0%, 85.1%, and 78.3% higher than those on the upper slope. At the lower slope position, the leaf nitrogen content in the sunny slope was 6.37 mg/g, and that in the shady slope was 4.86 mg/g, which was significantly higher by 31.1%. At the upper slope position, the leaf potassium content on the shady slope was 3.81 mg/g, and that on the sunny slope was 2.21 mg/g, which was 72.4% higher.

Figure 3 also shows that there is no significant difference in leaf stoichiometry between shady and sunny slopes. In the down slope position, the N/K ratio of *S. mukorossi* leaves on the sunny slope was significantly higher than that on the shady slope by 60.8%, and in the middle and upper slope positions, it was extremely significantly higher by 81.1 and 66.7% (*p* < 0.01). In the middle slope position, the K/P ratio of leaves on the shady slope was 39.3% higher than that on the sunny slope.

### 2.4. Effects of Slope Aspect and Position on Soil Stoichiometry of S. mukorossi

**Figure 4:**
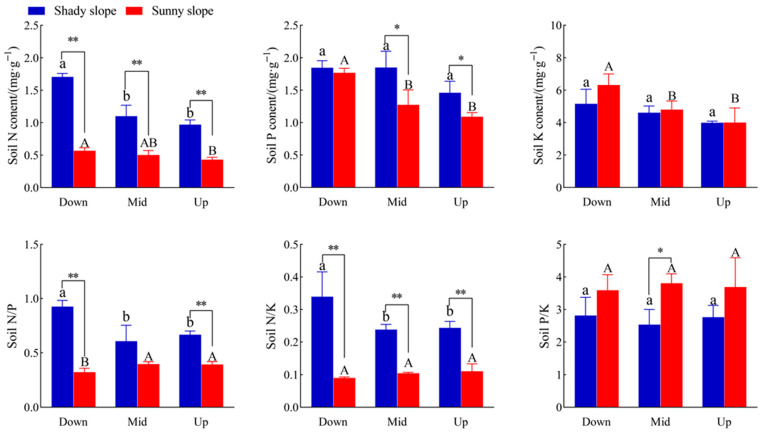
Effects of slope aspect and position on soil stoichiometry in *S. mukorossi.* Note: different lower case letters in the figure indicate significant difference between different slope positions on the shady slope (*p* < 0.05), and different majuscule indicate significant difference between different slope positions on the sunny slope (*p* < 0.05); * indicates significant difference between different aspects in the same slope position (*p* < 0.05), ** indicates extremely significant difference between different aspects in the same slope position (*p* < 0.01).

From Figure 4, the soil N content of *S. mukorossi* on the shady slope was the highest (1.71 mg/g) at the down slope position, which was significantly higher than that at the middle and upper slope positions by 55.5% and 76.3% (*p* < 0.05), respectively; however, soil P and K contents of *S. mukorossi* on the shady slope did not change significantly with the change in slope position (*p* > 0.05). The highest N, P, and K contents were 0.57, 1.77, and 6.32 mg/g in the down slope position, while the N content was significantly higher than that in the upper slope position by 32.6%, the P content was significantly higher than that in the middle and upper slope positions by 39.4 and 62.4%, respectively, and the K content was 31.7% and 58.0% higher than that in middle and upper slope positions, respectively. In the down slope position, the soil N content on the shady slope was significantly higher than that on the shady slope by 200%. In the middle slope position, the soil N content on the shady slope was significantly higher than that on the sunny slope by 120%, and the soil P content was significantly higher by 45.7%. In the upper slope position, the soil N content of the shady slope was significantly higher than that of the sunny slope by 125.6%, and the soil P content was significantly higher by 33.9%. Slope aspect had no significant effect on soil K content.

Figure 4 also shows that the N/P ratio of *S. mukorossi* on the shady slope is the highest at the down slope position, which is significantly higher by 52.5% and 38.8% than that at the middle and upper slope positions (*p* < 0.05), while the N/P ratio of *S. mukorossi* on the sunny slope is the lowest at the down slope position and soil N/P on the shady slope was 190.6% and 71.8% higher than that on the sunny slope (*p* < 0.01). The soil N/K on the shady slope was also the highest in the down slope position, which was 43.5% and 41.7% higher than that in the middle slope position and the upper slope position, respectively, while soil N/K on the shady slope was 277.8, 130.0, and 118.2% higher than that on the sunny slope. With the change in slope position, there was no significant difference in soil K/P among different slope aspects. In the middle slope position, soil K/P on the sunny slope was 50.2% higher than that on the shady slope.

### 2.5. Variation Characteristics of Various Indicators of S. mukorossi with Different Slope Aspects

The coefficient of variation (CV) is used to measure the degree of dispersion of data; the larger the coefficient, the greater the degree of dispersion of data. When CV ≤ 10%, it can be considered that the variation in the data is small and belongs to the range of weak variation. When CV is between 10% and 100%, it belongs to the range of medium variation, and strong variation refers to a coefficient of variation ≥ 100%. From Table 1, all indexes of *S. mukorossi* on the shady slope had moderate variation except DBH and leaf K/P. Except for leaf N/K, the other indexes of *S. mukorossi* on the sunny slope had moderate variation.

### 2.6. Phenotypic Plasticity Indices for Different Slope Aspects

**Figure 5:**
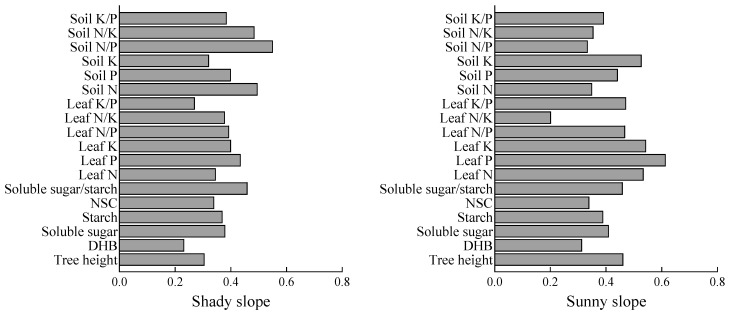
Phenotypic plasticity of indicators.

Figure 5 shows that the plasticity index of the shady slope is 0.23–0.55, and that of the sunny slope is 0.20–0.61. The plasticity indexes of soil N/P, soil N, soil N/K, and soluble sugar/starch on the shady slope were higher than those of DBH and leaf K/P. The plasticity indexes of leaf P, leaf K, leaf N, and soil K were higher on the sunny slope, and the plasticity index of leaf N/K was lower. The results showed that soil N/P, soil N, soil N/K, soluble sugar/starch, leaf P, leaf K, leaf N, and soil K had strong plasticity under different slope aspects.

### 2.7. Correlation of Various Indicators of S. mukorossi with Different Slope Aspects

**Figure 6:**
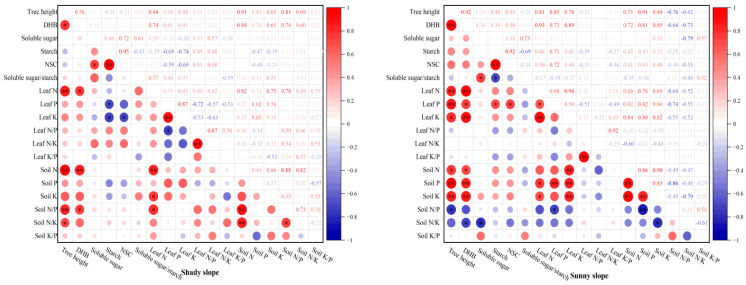
Correlation between *S. mukorossi* and soil stoichiometry with different slope aspects. Note: * indicates *p* < 0.05, ** indicates *p* < 0.01.

Figure 6 shows that the height of *S. mukorossi* on the shady slope was significantly positively correlated with DBH and soil N/K (*p* < 0.05), and was significantly positively correlated with leaf N, soil N, and soil N/P (*p* < 0.01). DBH was significantly positively correlated with leaf N and soil N/P, and was significantly positively correlated with soil N. There was a significant positive correlation between soluble sugar and NSCs. Starch was positively correlated with NSCs and negatively correlated with leaf P and K (*p* < 0.05). NSCs were negatively correlated with leaf K. Leaf N was significantly positively correlated with soil N, soil K, and soil N/P. Leaf N/P was positively correlated with leaf N/K. Soil N was positively correlated with soil N/P and soil N/K. Soil N/P was positively correlated with soil N/K. The above results showed that there were different degrees of correlation between the indexes of *S. mukorossi* on the shady slope.

Figure 6 also shows that the height of *S. mukorossi* on the sunny slope is significantly positively correlated with DBH, leaf N, leaf P, soil P, and soil K, and is significantly positively correlated with leaf K and soil N, but was negatively correlated with soil N/P. DBH was positively correlated with leaf N, leaf K, soil P, and soil K, negatively correlated with leaf P and soil N, and negatively correlated with soil N/K. Soluble sugar was positively correlated with soluble sugar/starch and negatively correlated with soil N/K. Starch was positively correlated with NSCs, negatively correlated with soluble sugar/starch, and positively correlated with leaf P. NSCs were positively correlated with leaf P. Leaf N was significantly positively correlated with leaf P, soil P, and soil K, and very significantly positively correlated with leaf K. Leaf P was positively correlated with soil P and negatively correlated with soil N/P. Leaf K was significantly positively correlated with soil N, soil P, and soil K. Leaf N/P was positively correlated with leaf K/P. Soil N was significantly positively correlated with soil P and soil K. Soil P was positively correlated with soil K and negatively correlated with soil N/P. Soil K was significantly negatively correlated with soil N/K. The above results show that there are different degrees of correlation between the indices of the sunny slope.

## 3. Discussion

### 3.1. Effects of Slope Aspect and Position on Tree Height and DBH of S. mukorossi

Different site conditions directly affect light, temperature, moisture, and soil texture and other factors [24]. Tree height and DBH are the most basic morphological characteristics of tree growth and development, which can reflect the adaptability of trees to external environmental changes [7]. In the present study, it was found that for the same slope orientation, both the height and diameter at breast height of *S. mukorossi* reached their maximum at the down slope position, and at the middle slope position, the height of trees at the shady slopes was highly significantly higher than that of the sunny slopes. In the upper slope position, the height of trees on the shady slopes was also significantly higher than that on the sunny slopes, and the diameter at breast height (DBH) was significantly higher than that on the sunny slopes. It showed that *S. mukorossi* grew better with the same slope aspect and down slope position and grew better in the same slope position and shady slope position. In this study, the tree height and DBH of *S. mukorossi* on sunny and shady slopes were the largest on the down slope position and the smallest on the upper slope position, which were significantly lower than those on the down slope position. This is similar to the findings of Liu Xin et al. with *Populus simonii* [25]. This may be due to the influence of slope position on environmental factors such as soil, water, and nutrients, which leads to the fact that the down slope position provides superior growth conditions for *S. mukorossi* due to soil fertility, sufficient water, and nutrient enrichment. Aspect is one of the topographic factors that plays a key role in the growth of plants [26]. In this study, in the middle slope position, the height of *S. mukorossi* on the shady slope was significantly higher than that on the sunny slope; in the upper slope position, the height and DBH of *S. mukorossi* on the shady slope were significantly higher than those on the sunny slope. This may be due to the fact that sunny slope’s light intensity, long duration, evaporation, and low soil moisture are not conducive to tree growth [27]. This study also found that there was no significant difference in the growth of tree height and DBH between the shady slope and the sunny slope at the down slope position. This may be due to sufficient soil water and nutrients and more balanced resource allocation in the down slope position, which is not enough to trigger the growth response of *S. mukorossi*. Of course, the change in microclimate and the difference in soil texture also have a certain influence on tree height and breast diameter.

### 3.2. Effects of Different Slope Aspect and Position on Leaf NSC Characteristics of S. mukorossi

The changes in soluble sugar and starch content in plants and their mutual transformation relationship are important physiological responses of plants to cope with the external environment and maintain their life activities [28]. In this study, the soluble sugar content of *S. mukorossi* leaves did not change significantly with slope aspect and slope position, while the starch content of *S. mukorossi* leaves on the shady slope was the highest at the middle slope position and the content of starch in leaves of *S. mukorossi* on the sunny slope was the highest on the upper slope. This may be due to the direct involvement of soluble sugars in plant physiological activities, which are the main forms of carbohydrate transport and utilization, and their functions that can support plant growth and development under different external environments [29]. Therefore, in this study, soluble sugar has a certain degree of stability. The results showed that under the same slope aspect, different slope positions had a greater impact on the starch content of *S. mukorossi* leaves. The change in NSC content in plants reflects the balance state of the carbon budget of trees, which is helpful to understand the change law of plant growth and carbon allocation and the adaptation strategy to environmental stress [30]. In this study, on the upper slope, the starch content of leaves on the shady slope was significantly higher than that on the sunny slope, and the NSCs of leaves was significantly higher than that on the sunny slope. Combined with the height and DBH of *S. mukorossi*, it is possible that the NSCs produced by *S. mukorossi* is mainly used for growth because of the good water conditions, shaded conditions, and less transpiration on the shady slope. This is similar to the results of Liu et al. [31].

### 3.3. Effects of Different Slope Directions and Slope Positions on Leaf–Soil Stoichiometric Characteristics of S. mukorossi

Leaf stoichiometry is associated with plant growth and development and many key functions in ecosystems and serves as an indicator to evaluate plant nutrient use and response to environmental changes [15,32]. N and P are essential nutrients for plant growth, and changes in their content play a key role in regulating plant growth [33]. At the same time, soil N and P elements limit plant growth and development and improving the absorption and utilization efficiency of both nutrients is an important nutrient regulation mechanism for plants to adapt to different soil conditions [34]. In this study, the leaf and soil n contents of *S. mukorossi* on shady and sunny slopes were the highest on the down slope and the lowest on the upper slope. This may be due to strong environmental stress in the upper slope position, where the soil nutrients in the upper slope position flow to the down slope position due to surface runoff and other reasons, and the plants in the upper slope position cannot obtain enough n to meet their growth needs, resulting in a lower leaf and soil n content. This study also found that the leaf n content of *S. mukorossi* on the sunny slope was significantly higher than that on the shady slope at the down slope position, and the soil n content on the shady slope was extremely significantly higher than that on the sunny slope, which may be due to the larger environmental stress of the plants on the sunny slope as plants respond to environmental stress by increasing their N content. The sunny slope also receives more solar radiation and experiences a higher temperature and more water evaporation, which is not conducive to the accumulation and maintenance of soil nutrients. In this study, the leaf and soil P contents of *S. mukorossi* on the sunny slope were the highest in the down slope position and the lowest in the upper slope position, while there was no significant difference in leaf and soil p contents of *S. mukorossi* on the shady slope with slope position. This may be due to the difference in slope position. With the rise in slope position, soil erosion and nutrient loss are greater, the erosion effect of surface runoff on soil is enhanced, and the P element in the soil is reduced in the upper slope position, which at the same time leads to a decrease in the P uptake by plants in the soil [35]. The average content of total potassium in soil in China is 16.6 g/kg and the average level of K in leaves of terrestrial plants in China is 15.09 g/kg [36,37]. In this study, the K content in the leaves and soil of *S. mukorossi* was less than the average level with different slope aspects and slope positions. It may also be related to soil erosion and nutrient loss.

Soil ecological stoichiometry reflects not only the cycling and balance of soil nutrients, but also the nutrient status during soil development, making it an important indicator of soil quality [38]. In this study, soil N/P on the shady slope was significantly higher than that on the sunny slope in both the upper and down slope positions. This also shows that the sunny slope receives more solar radiation and experiences a higher temperature and greater water evaporation, which is not conducive to the accumulation and maintenance of nutrients in the soil, especially N, which is easy to lose in the form of gas. Plant leaf N/P can be used to illustrate the status of plants limited by N and P. When N/P is more than 16, plants are mainly limited by P; when N/P is less than 14, plants are mainly limited by N; and when N/P is between 14 and 16, they are limited by both elements [39,40]. When N/K > 2.1 and K/P < 3.4, plant growth is mainly restricted by K [41]. In this study, the N/P of *S. mukorossi* leaves in different slope positions on the shady slope was <14, N/K was <2.1, and K/P was >3.4, indicating that the growth of *S. mukorossi* on the shady slope was mainly limited by N, not K. On the sunny slope, N/P was less than 14, N/K was less than 2.1, but K/P was less than 3.4, which indicated that the growth of *S. mukorossi* on the sunny slope was also limited by N, and whether it was limited by K needs further experiments to be proved. Combined with local fertilization conditions, N fertilizer is the main fertilizer. Seasonal variation, plant developmental stage, and physiological state are all factors that significantly affect N/P and N/K ratios. Nutrient stress itself is a dynamic process, but is also involved in plant uptake, soil release, seasonal changes, plant development stage, microbial activities, and other factors; it is suggested that further studies or fertilization experiments should be carried out in the future. It was also found that the soil N content on the shady slope was significantly higher than that on the sunny slope at the same slope position. Combined with the height and DBH growth of *S. mukorossi* (Figure 1), it has been shown that *S. mukorossi* grew better on the shady slope.

### 3.4. Variation Characteristics, Plasticity, and Correlation Analysis of S. mukorossi Plantations with Different Slope Aspects

Leaves are the main photosynthetic organs of plants and the energy source organs of trees, and the response of plants to environmental changes is often concentrated in the leaves [14]. Soil is the basis for plant growth and the developmental needs of water, fertilizer, gas, heat, and other ecological elements are provided through the soil [42]. In this study, all the indexes of *S. mukorossi* on the shady slope exhibited moderate variation except for DBH and leaf K/P, which exhibited weak variation. In addition to the weak variation in leaf N/K, the other indexes of *S. mukorossi* on the sunny slope also exhibited moderate variation. This shows that *S. mukorossi* can adapt to the environmental conditions of different slope aspects to a certain extent. Although there is a certain degree of variation, the coefficient of variation is moderate and below. The results of plasticity index showed that under different slope aspects, soil N/P, soil N, soil N/K, soluble sugar/starch, leaf P, leaf K, leaf N, and soil K had strong plasticity. This study also found that there were different degrees of correlations among the growth of *S. mukorossi*, leaf NSCs and its components, and leaf–soil stoichiometry with different slope aspects. This may be because soil nutrients can directly drive changes in stoichiometry in plants, affecting plant growth [43]. Plants absorb nutrients from the soil to synthesize organic matter, on the one hand, to supply their own needs, and on the other hand, to return to the soil in the form of senescent leaves, thus forming a correlation between the plant–soil nutrient cycle [44]. However, this correlation may also be affected by plant growth stage, the external environment, and other factors.

## 4. Materials and Methods

### 4.1. General Situation of the Test Site

The experimental site was located at the Chinese *S. mukorossi* germplasm resources monitoring base of the National Innovation Alliance of *S. mukorossi* Industry in Zhenfeng, Guizhou Province (25°12′20″ N, 105°52′25″ E), which is 369–1165 m above sea level. The area has a subtropical monsoon humid climate with an annual rainfall of 1356.7 mm, annual average temperature of 23 °C, yellow soil type, and planting density of 3 m × 3 m.

### 4.2. Design of Experiment

According to the actual terrain, the slope is divided into upper, middle, and lower parts. The horizontal distance of each part is about 200 m, and the same slope position of each slope position is on the same contour line. The sample plots of different site conditions were set to 20 m × 20 m, and each tree in each sample plot was checked for growth indicators. There are about 45 *S. mukorossi* trees in each plot. In each sample plot, 5 sampling points were selected using the diagonal method, and the microclimate factors and soil factors were observed.

### 4.3. Indicator Determination

On the 26 April 2024, the weather was sunny. The microclimate in the plot was measured and *S. mukorossi* was sampled. The tree height and DBH of each *S. mukorossi* were investigated in each plot. The tree height was measured using a laser altimeter. The DBH of a tree was measured with a diameter at DBH ruler. At the same height above the ground, light radiation was measured by portable illuminators (UT383s, UNI-T) at a fixed diagonal position in each plot, while soil temperature was measured with a portable soil thermometer and hygrometer (TDR300, Spectrum). The microclimate factors and soil factors are shown in Table 2. After removing the surface litter, the 0–20 cm surface soil was drilled with a soil drill and mixed into a soil sample, immediately put into a Ziplock bag, and taken back to the lab. Three standard trees were selected from each sample plot, and about 10–12 healthy mature leaves were collected using a tree-by-tree pruner. The collected leaf markers were loaded into envelopes and placed in an oven for 30 min at 120 °C. The oven was adjusted to 80 °C and the leaves were dried to a constant quality. The mortar was ground and crushed, passing through a 120-mesh sieve. The determination of soluble sugar and starch content was completed using phenol colorimetry, where non-structural carbohydrate content = soluble sugar content + starch content [45]. An ultraviolet spectrophotometer (MAPADA, UV-6100, Shanghai, China) was used in the determination process, and a centrifuge was set (4000 r/10 min). The total nitrogen content was determined by colorimetry, the total phosphorus content was determined by vanadium molybdenum yellow colorimetry, and the total potassium content was determined by flame photometry. The detailed method is shown in Bao Shidan, *Soil Agrochemical Analysis 3rd Edition*, China Agriculture Press [46].

Standard curve making: Take a 20 mL test tube numbered from 0 to 10, use Table 3 and Table 4 in turn to add solution and water, and then in turn, according to the number, add 1 mL 9% phenol solution to the test tube, shake, add 5 mL concentrated sulfuric acid every 5 s to 20 s from the top of the tube, shake well, until the total volume of the colorimetric solution is 8 mL, and leave at room temperature for 30 min for color development. It was then analysed colorimetrically at 485 nm using a UV–visible photometer (preheated for 15 min before use) with a blank as a reference. The standard curve equation was obtained with the soluble sugar content as the abscissa and the optical density as the ordinate. The method of adding reagents to the production of a starch standard curve is consistent with the method of soluble sugar.

Preparation of main reagents:

① 90% phenol solution: use analytical balance to weigh 90 g phenol (AR), add 10 mL distilled water to dissolve, mix well, and put it into reagent bottle cap and label.

② 9% phenol solution: the volume ratio of 90% phenol solution to distilled water is 1:9.

③ 1% sucrose standard solution: the pure sucrose will be analyzed at 80 °C under constant weight, and 1.000 g was taken accurately. Dissolve in a small amount of water, transfer to a 100 mL volumetric flask, add 0.5 mL of concentrated sulfuric acid, bring to volume with distilled water to scale, and mix well.

④ 100 ug/mL sucrose standard solution: accurately absorb 1 mL 1% sucrose standard solution into 100 mL volumetric flask, add water to volume, and shake well.

⑤ 9.2 mol/HCI04 solution: to prepare 100 mL solution, absorb 70% HC104 solution 36.2 mL in 100 mL volumetric flask, then add distilled water to scale, shake, and pour into the reagent bottle to label and store.

⑥ 100 ug/mL starch standard solution: accurately weigh 100 mg pure starch, put into 100 mL volumetric flask, add 60–70 mL hot distilled water, boil in boiling water bath for half an hour; after cooling, add distilled water dilution to scale, and shake. Then draw 5 mL solution into 50 mL volumetric flask, add distilled water to scale, shake, and then it is ready to use.

Nesslerization: Nessler’s reagent is 0.09 mol/L of potassium mercuric iodide mixed with 2.5 mol/L of potassium hydroxide. Determination of N content based on UV–visible spectrophotometry.

Weigh 0.2 g of the sample, add 1.5 mL of distilled water to moisten it, and mix thoroughly. Then add 5 mL of concentrated sulfuric acid and shake well. Place the mixture in a digestion vessel for boiling. Before introducing it into the digestion furnace, add 5 drops of H_2_O_2_. When the furnace reaches 120 °C, add another 5 drops of H_2_O_2_ and boil for 30 min. When the temperature reaches 180 °C, add 5 drops of H_2_O_2_ and continue boiling for 30 min. When the temperature reaches 240 °C, add 5 drops of H_2_O_2_ and boil for 30 min. Finally, when the temperature reaches 300 °C, add 5 drops of H_2_O_2_ until the mixture becomes clear. After clarification, allow it to cool naturally before adjusting the volume.
2.5 mL digested liquid to be tested↓1 mL sodium tartrate↓2.7 mL KOH↓Add water to 20 mL↓Nessler’s reagent 1.25 mL↓Add water to 25 mL↓30 min of color development, spectrophotometer for 420 nm determination

### 4.4. Data Processing and Analysis

Excel 2016 and SPSS 27.0 were used for experimental data processing and analysis. A one-way ANOVA and independent sample t-test were used and the tree height and DBH, leaf non-structural carbohydrates, and leaf–soil stoichiometry of *S. mukorossi* plantation with different slope aspects and positions were analyzed (*p* < 0.05, *p* < 0.01). Duncan’s method was used for multiple comparisons, provided the data met the normality test and variance homogeneity tests. Pearson’s correlation analysis was used to analyze the correlation between each index (*p* < 0.05, *p* < 0.01). The indicators were plotted in Graphpad prism 9.5 and Origin 2024.

Plasticity index (P): P = (X*max* − X*min*)/X*max*

Coefficient of variation (CV): CV = Standard deviation/average × 100%

## 5. Conclusions

By studying the effects of different slope directions on the tree height and DBH, leaf NSC characteristics, and leaf–soil stoichiometric ratio of *S. mukorossi*, it was found that the height and DBH of *S. mukorossi* in the down slope position grew better in the same slope direction, while the height and DBH of *S. mukorossi* on the shady slope grew better in the same slope position. There was no significant difference in the soluble sugar in leaves of *S. mukorossi* with the change in slope position and slope direction. On the upper slope, the starch in leaves of the shady slope was significantly higher than that of the sunny slope, and NSCs were significantly higher than that of the sunny slope. The leaf and soil n contents of *S. mukorossi* on shady and sunny slopes were the highest on the upper slope and the lowest on the down slope. Combined with the height and DBH of *S. mukorossi*, the shady slope was more suitable for the growth of *S. mukorossi*. The leaf and soil P contents of *S. mukorossi* on the sunny slope were the highest in the down slope position and the lowest in the upper slope position, while there was no significant difference in leaf and soil p contents of *S. mukorossi* on the shady slope with slope position. The K content in the leaves and soil of *S. mukorossi* on different slopes was less than the average K content in the leaves of terrestrial plants in China. The growth of *S. mukorossi* on the shady slope was mainly limited by N, and it was also limited by N on the sunny slope. To a certain extent, *S. mukorossi* can adapt to the environmental conditions on different slopes. Although there is a certain degree of variation, the coefficient of variation is medium and below. With different slope aspects, soil N/P, soil N, soil N/K, soluble sugar/starch, leaf P, leaf K, leaf N, and soil K had strong plasticity. The tree height and DBH of *S. mukorossi*, leaf NSCs and its components, and leaf–soil stoichiometry were correlated to different degrees on different slopes. In the future, researchers can further study the microbial community, root–soil interaction, and seasonal variation.

## Figures and Tables

**Table 1:** Variation characteristics of indexes of *S. mukorossi* with different slope aspects.

Slope	Indicators	Mean	SD	Value Range	CV/%
Shady slope	Tree height	7.14	0.94	5.9–8.5	13
	DHB	7.51	0.66	6.6–8.6	9
	Soluble sugar	30.10	5.35	23.10–37.29	18
	Starch	76.32	11.44	60.14–94.90	15
	NSCs	106.41	14.68	86.10–131.43	14
	Soluble sugar/starch	0.40	0.07	0.29–0.53	17
	Leaf N	4.21	0.60	3.64–5.57	14
	Leaf P	0.97	0.18	0.72–1.28	18
	Leaf K	4.38	0.66	3.2–5.34	15
	Leaf N/P	4.43	0.77	3.31–5.46	17
	Leaf N/K	0.97	0.14	0.73–1.17	15
	Leaf K/P	4.56	0.40	3.92–5.37	9
	Soil N	1.26	0.35	0.89–1.77	28
	Soil P	1.72	0.25	1.26–2.10	15
	Soil K	4.59	0.71	3.9–5.75	15
	Soil N/P	0.73	0.17	0.44–0.98	23
	Soil N/K	0.27	0.06	0.22–0.43	23
	Soil K/P	2.70	0.43	2.00–3.25	16
Sunny slope	Tree height	6.15	1.46	4.4–8.2	24
	DHB	7.31	0.98	5.9–8.6	13
	Soluble sugar	32.48	5.06	22.81–38.90	16
	Starch	71.86	11.60	56.14–92.05	16
	NSCs	104.34	12.55	82.48–124.29	12
	Soluble sugar/starch	0.46	0.11	0.35–0.65	24
	Leaf N	5.05	1.30	3.06–6.59	26
	Leaf P	0.97	0.29	0.60–1.56	30
	Leaf K	3.09	0.85	1.96–4.29	28
	Leaf N/P	5.37	1.17	3.78–7.11	22
	Leaf N/K	1.64	0.15	1.50–1.87	9
	Leaf K/P	3.29	0.73	2.15–4.07	22
	Soil N	0.50	0.07	0.39–0.60	15
	Soil P	1.38	0.33	1.02–1.83	24
	Soil K	5.04	1.20	3.23–6.85	24
	Soil N/P	0.37	0.04	0.28–0.42	12
	Soil N/K	0.10	0.01	0.09–0.14	14
	Soil K/P	3.69	0.54	2.79–4.60	15

**Table 2:** Climate factors of the test site.

Slope Aspect	Slope Position	Soil Temperature (°C)	Wind Speed (m/s)	Illuminance (lx)
Sunny slope	Up	35.6	0.417	1072 × 100
	Mid	36.8	0.413	1012 × 100
	Down	37.3	0.433	1035 × 100
Shady slope	Up	37.1	1.000	1128 × 100
	Mid	36.4	1.375	1114 × 100
	Down	36.8	0.658	1142 × 100

**Table 3:** Sample quantity added in the preparation of standard curve for soluble sugar.

Tube Number	100 ug/mL Sucrose Standard Solution (mL)	Distilled Water (mL)	Sugar Content (μg)
0	0.00	2.00	0
1–2	0.20	1.80	20
3–4	0.40	1.60	40
5–6	0.60	1.40	60
7–8	0.80	1.20	80
9–10	1.00	1.00	100

**Table 4:** Sample quantity added in the preparation of standard curve for starch.

Tube Number	100 ug/mL Starch Standard Solution (mL)	Distilled Water (mL)	Starch Content (μg)
0	0.00	2.00	0
1–2	0.40	1.60	40
3–4	0.80	1.20	80
5–6	1.20	0.80	120
7–8	1.60	0.40	160
9–10	2.00	0.00	200

## Data Availability

Data are contained within the article.
